# Genome-wide analysis of basic helix-loop-helix superfamily members in peach

**DOI:** 10.1371/journal.pone.0195974

**Published:** 2018-04-16

**Authors:** Chunhua Zhang, Ruchao Feng, Ruijuan Ma, Zhijun Shen, Zhixiang Cai, Zhizhong Song, Bin Peng, Mingliang Yu

**Affiliations:** Institute of Pomology, Jiangsu Academy of Agricultural Sciences/Jiangsu Key Laboratory for Horticultural Crop Genetic Improvement, Nanjing, Jiangsu, China; Wuhan Botanical Garden, CHINA

## Abstract

The basic helix-loop-helix (bHLH) transcription factor family is a superfamily found in all eukaryotes that plays important roles in regulating growth and development. Over the past several decades, many *bHLH* superfamily genes have been identified and characterized in herbaceous and woody plants. However, the genes belonging to the bHLH superfamily in peach (*Prunus persica*) have not yet been comprehensively identified and characterized. Here, we identified 95 members of the bHLH superfamily in the peach genome, and these genes were classified into 19 subfamilies based on a phylogenetic comparison with bHLH proteins from *Arabidopsis*. The members within each subfamily were highly conserved according to the analysis of motif compositions and exon/intron organizations. The 95 *bHLH* genes were unevenly distributed on chromosomes 1 to 8 of the peach genome. We identified 57 pairs of bHLH members that were orthologous between peach and *Arabidopsis*. Additionally, 48 pairs of paralogous *bHLH* genes were identified on the eight chromosomes of the peach genome. Coupled with relative expression analysis of *bHLH* genes in red-fleshed peach fruit at five developmental stages, we identified several *bHLH* genes that might be involved in fruit development and anthocyanin biosynthesis. This study provides insight into the molecular mechanisms through which these genes are involved in the regulation of biological and biochemical processes in peach and lays the foundation for further studies on these genes.

## Introduction

Transcription factors (TFs) play key roles in various physiological and biochemical processes in different tissues at different developmental stages in plants, mainly by repressing or activating related downstream genes to regulate gene expression, thereby controlling the growth, development, and stress response of plants [1,2]. The basic helix-loop-helix (bHLH) superfamily is considered the second largest TF family and is found across eukaryotic kingdoms. Proteins of the bHLH superfamily in all eukaryotic organisms are characterized by a highly conserved bHLH domain, which is approximately 50–60 amino acids in length and is divided into two distinctive regions: the basic region and the HLH region [3,4]. The basic region, which is approximately 10–15 predominant amino acids in length and is located at the N-terminus of the domain, is the DNA-binding interface and is required for DNA binding. The HLH region, which contains two amphipathic α-helices connected by a loop of variable length, acts as dimerization domain [1]. In addition to this highly conserved bHLH domain, other conserved motifs also exist within some bHLH subfamilies of the bHLH superfamily [5,6]. Based on conserved domains and phylogenetic relationships, the bHLH superfamily is usually classified into 15–25 subfamilies in plants [6,7]. As an increasing number of genome sequences are being released, a variety of *bHLH* superfamily genes have been identified and analyzed in a wide range of plant species, such as peanut [8], tomato [9], apple [10], blueberry [11], *Arabidopsis* [12], and Chinese cabbage [13]. Furthermore, the functions of many bHLH proteins in plants have been studied in detail. The results from these studies have shown that these proteins are involved in the regulation of various networks or biological and biochemical processes throughout the plant life cycle, including Fe uptake [14], tanshinone biosynthesis [15], petal growth [16], and the response to drought and salt stress [10,17]. Most importantly, many studies have suggested that bHLH proteins are involved in the regulation of fruit development and in anthocyanin biosynthesis in both flowers and fruits [18–21].

Peach (*Prunus persica*) is a diploid model plant (2n = 2x = 16) of the Rosaceae family and has a relatively small genome size of 265 Mb [22]. Flesh color is an attractive and important trait for the commercial value of peaches. In general, peaches can exhibit four different flesh colors: white, green, yellow and red. Anthocyanin has been reported to contribute to red flesh color, and *bHLH* genes have been suggested to be involved in the biosynthesis of anthocyanin in some fruit species. Recently, whole-genome sequences of peach were added to the Genome Database for Rosaceae (GDR), providing an important foundation for the genome-wide identification of genes within a given peach family. To the best of our knowledge, genes of the peach bHLH superfamily have not been comprehensively identified and characterized to date. The aims of this study were as follows: (1) identification and characterization of peach *bHLH* superfamily genes; (2) subfamily classification of peach *bHLH* superfamily genes; (3) determination of the chromosomal distribution of peach *bHLH* genes and their syntenic relationships with *Arabidopsis*; and (4) expression profiling of *bHLH* genes via qRT-PCR analyses. The findings of this study will be helpful for future functional identification of specific *bHLH* superfamily genes involved in anthocyanin biosynthesis and fruit development in peach.

## Materials and methods

### Plant materials

The fruits from one-year-old fruiting shoots were selected as the experimental materials from the outer southern canopies of six-year-old ‘Zhaoxia’ (ZX, white flesh) and ‘Yejihong’ (YJH, red flesh) peach trees grown under standard field conditions at the National Peach Germplasm Repository in Nanjing, China. The fruits from the two above-mentioned cultivars were sampled 51, 64, 75, 84, and 93 days after full bloom (DAFB), immediately frozen in liquid nitrogen and stored at -80°C.

### Identification of peach *bHLH* superfamily genes

The hidden markov model (HMM) file of the HLH domain (PF00010) was downloaded from the Pfam database (version 30.0; http://pfam.xfam.org/) [23] and was used as a query to scan the peach genome using HMMER software (version 3.1b2; http://hmmer.org/) with a default E-value. The protein sequences for the candidate genes with ID numbers identified from the HMMER results were downloaded from the Phytozome database (https://phytozome.jgi.doe.gov). These protein sequences were further analyzed with the online CD-search tool (https://www.ncbi.nlm.nih.gov/) and InterProScan (http://www.ebi.ac.uk/Tools/InterProScan/) to verify the existence of the conserved bHLH domain. Redundant sequences were removed manually.

### Motif identification and phylogenetic analysis of bHLH proteins

The online tool Multiple EM for Motif Elicitation (MEME, version 4.8.1) was used to search for conserved motifs among bHLH proteins (http://meme.nbcr.net/meme/cgi-bin/meme.cgi) by uploading the amino acid sequences of the peach bHLH superfamily proteins. The parameter settings were as follows: 0 or 1, occurrence of a single motif per sequence; 2 to 50 amino acids, motif width range; and 3, maximum number of motifs identified. All other parameters were set at the default values. Multiple sequence alignment of peach bHLH proteins (amino acid sequences) was conducted using the ClustalW program [24]. The amino acid sequences and chromosomal location of the 161 bHLH superfamily members of *Arabidopsis* were downloaded from The Arabidopsis Information Resource (TAIR) database (http://www.arabidopsis.org/). Phylogenetic trees of the bHLH superfamily proteins from *Arabidopsis* and peach were constructed with the ClustalW tool in conjunction with MEGA 4.1 software [24] using the neighbor-joining method and 1000 bootstrap replicates. Based on the subfamily classification of the bHLH proteins from *Arabidopsis*, the phylogenetic tree of peach and *Arabidopsis* constructed in this study, and the characteristics and structures of the genes and proteins, the peach bHLH superfamily was classified into subfamilies.

### Analysis of the characteristics and structures of *bHLH* genes

The genomic sequences, ID numbers (peach v1.0 and v2.0), coding sequences (CDSs), transcript sequences, and genomic locations of peach *bHLH* genes were downloaded from the Phytozome database (https://phytozome.jgi.doe.gov/pz/portal.html#). The distribution of each gene on the eight chromosomes of peach was visualized using the circlize software package in R. The start and end positions of each *bHLH* member on each chromosome in *Arabidopsis* were obtained from the TAIR database. The orthologous *bHLH* genes between peach and *Arabidopsis* as well as the paralogous genes in peach or *Arabidopsis* were predicted using OrthoMCL software (http://orthomcl.org/orthomcl/), and synteny figures were then drawn using the Circos tool (http://mkweb.bcgsc.ca/circos). The structural features of the peach *bHLH* genes, including exon and intron numbers as well as locations, were analyzed using the Gene Structure Display Server (GSDS) online tool (http://gsds.cbi.pku.edu.cn/).

### Expression analysis of peach *bHLH* superfamily genes

The total RNA from fruits sampled from the two cultivars was extracted using the Plant RNA Kit (TaKaRa Biotechnology Co. Ltd., Dalian, China). The extracted RNA was then reverse transcribed to cDNA using the PrimeScript^™^ RT Reagent Kit (TaKaRa Biotechnology Co. Ltd., Dalian, China). All cDNA samples were diluted to 100 ng μl^−1^ with RNase-free water and stored at -20 °C before being used as templates for quantitative real-time PCR (qRT-PCR).

Gene-specific primers for qRT-PCR of the 22 *bHLH* genes were designed based on the CDSs of the *bHLH* genes using Primer Premier 5.0 software (Premier Biosoft) (S1 Table). To guarantee primer specificity, one of each pair of primers was designed to not be located within the conserved domain. *RNA polymerase II* (*RP II*, accession number TC1717) and *translation elongation factor 2* (*TEF2*, accession number TC3544) of peach were used as the reference genes for qRT-PCR. qRT-PCR was conducted on an Applied Biosystems 7500 real-time PCR system using SYBR^®^ Premix Ex Taq^™^ reagent (Tli RNaseH Plus) (TaKaRa Biotechnology Co. Ltd., Dalian, China) according to the manufacturer’s instructions. Each 20-μl reaction contained 1.0 μl of diluted cDNA, 0.4 μl of each primer, 0.4 μl of ROX, 10.0 μl of master mix, and 7.8 μl of RNase-free water. Thermal cycling conditions were set as per the manufacturer’s instructions for SYBR^®^ Premix Ex Taq^™^. Each assay was repeated three times using replicate fruit samples. The relative expression levels of each gene were calculated using the 2^-ΔΔ*C*^_T_ method [25].

## Results and discussion

### Identification of peach *bHLH* superfamily genes

Based on the HMM results, a total of 95 genes belonging to the peach bHLH superfamily were identified in this study (S2 Table). To verify the reliability of each member, the 95 protein sequences were analyzed with the online CD-search tool and InterProScan, and all 95 bHLH proteins were found to exhibit a typical bHLH domain. To a certain degree, this finding is consistent with the fact that the bHLH superfamily is the second largest gene family in plants. Compared with the number of *bHLH* genes identified in other plant species in previous studies, fewer *bHLH* superfamily genes were identified in peach in this study. In previous studies, 132, 124, 188, 225, 155, 167, 146 and 159 *bHLH* genes were identified in peanut [8], potato [26], apple [10], wheat [27], bean [17], rice [4], carrot [28], and tomato [9], respectively. These values are probably associated with differences in evolution and genome duplication or genome sizes in these plants. The density of *bHLH* superfamily genes in the peach genome was approximately 0.36, which is lower than those in the genomes of *Arabidopsis thaliana* (1.11), Chinese cabbage (0.81), and papaya (0.65) but higher than those in the genomes of lower plants, including *Volvox carteri* (0.024) and *Chlorella vulgaris* (0.081) [13].

The ID numbers of the 95 peach *bHLH* genes from both peach genomes v1.0 and v2.0 are listed in S2 Table. The corresponding ID numbers and sequences for peach genome v1.0 were not found for the *Prupe*.*6G159200*.*1*, *Prupe*.*7G040100*.*1*, and *Prupe*.*1G540400*.*1* genes (ID numbers of peach genome v2.0) in the Phytozome and GDR databases (S2 Table). This disparity is a result of the lower quality of the peach v1.0 genome assembly compared with that of the peach v2.0 genome assembly. The peach v1.0 assembly was improved using a large amount of community molecular mapping data, resulting in the peach v2.0 assembly [29]. Previously, unmapped sequences (7.3 Mb) of the peach v1.0 assembly were integrated within the eight peach chromosomes in the peach v2.0 assembly [29]. The lengths of the CDSs, peptide sequences, genomic sequences, and transcript sequences were recorded and are shown in S2 Table. The lengths of the peptide sequences of the 95 peach *bHLH* genes ranged from 123 to 729 bp, the lengths of the CDSs of the 95 peach *bHLH* genes ranged from 372 to 2190 bp, and the lengths of the genomic sequences of the 95 peach *bHLH* genes ranged from 561 to 7530 bp. The predicted functions of these *bHLH* genes are also listed in S2 Table.

The best homolog of each peach *bHLH* gene in *Arabidopsis* was extracted from the TAIR database (S2 Table). Some peach *bHLH* genes shared the highest homology with the corresponding gene in *Arabidopsis*; for example, both *Prupe*.*5G144400*.*1* and *Prupe*.*1G217700*.*1* were homologous to *AT5G50915*.*2*, with E-values of 1e-47 and 3e-26, respectively. Other peach genes that shared an *Arabidopsis* homolog included *Prupe*.*6G022200*.*1* and *Prupe*.*7G071500*.*1* as well as *Prupe*.*8G143600*.*1* and *Prupe*.*1G424100*.*1*. We inferred that a duplication event for peach *bHLH* genes might have occurred during the evolution of development or resistance to stress.

### Phylogenetic analysis, motifs, and multiple sequence alignment of bHLH proteins

**Fig 1:**
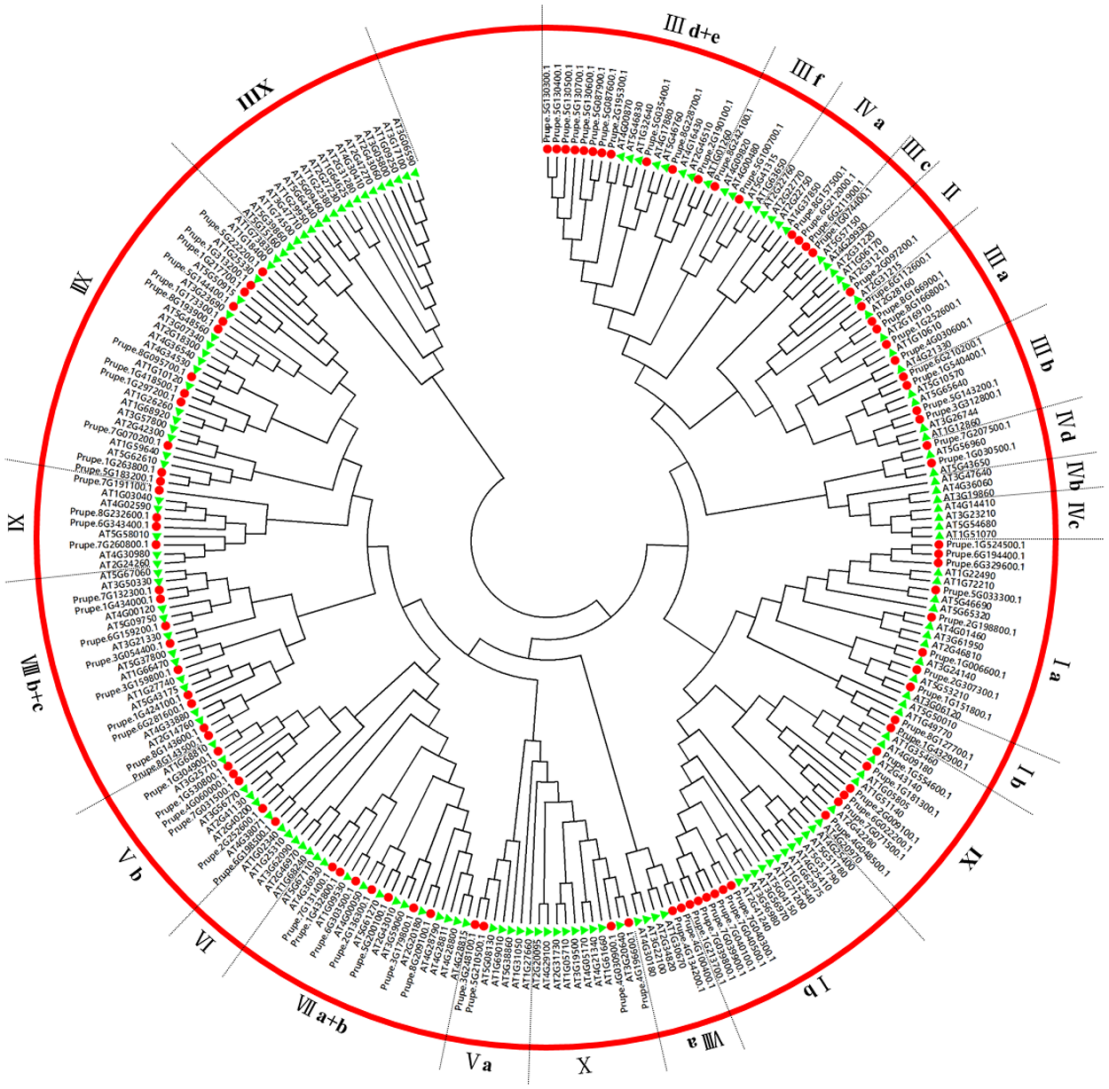
Phylogenetic tree constructed using the sequences of bHLH proteins from both peach and *Arabidopsis*. The small red circles represent the peach bHLH proteins; the small green triangles represent the bHLH proteins from *Arabidopsis*. The Roman numeral outside the large red circle indicates the name of each subfamily of the peach bHLH superfamily. The black dotted line represents the initial or final boundary of each subfamily of the peach bHLH superfamily.

To classify the peach bHLH proteins into subfamilies and identify the evolutionary relationships between the bHLH proteins of peach and *Arabidopsis*, a phylogenetic tree was constructed using the sequences of the 95 peach bHLH proteins and 161 *Arabidopsis* bHLH proteins (Fig 1). As a result, the 95 peach bHLH members were clustered into 19 subfamilies based on the topology of the tree and the classification of the bHLH superfamily in *Arabidopsis* and Chinese cabbage [13]. The 19 subfamilies were designated I a, I b, II, III a, III b, III c, III d+e, III f, IV a, IV d, V a, V b, VII a+b, VIII a, VIII b+c, IX, X, XI, and XII (Fig 1). Most of these subfamilies are common and are consistent with the subfamily classification reported in previous phylogenetic analyses in other plant species, such as tomato [9], peanut [8], Chinese cabbage [13], and wheat [27]. This finding indicated that the bHLH proteins in the conserved subfamilies among plant species may play a fundamental role during plant development and evolution. Additionally, this result is consistent with the earlier finding that the bHLH superfamily in plants can be divided into 15–25 subfamilies [6]. Some previous studies have named the bHLH subfamilies using English letters [4,10], whereas most published reports have named them using Roman numerals [13,9,11,8,27,26,6], and some authors have named each subfamily using Arabic numerals [3,28]. For the peach bHLH subfamilies identified in this study, we adopted the nomenclature using Roman numerals, as employed for Chinese cabbage by Song et al. [13] and for other plants by Pires and Dolan [6]. None of the peach bHLH proteins were grouped into subfamilies IV b, IV c, VI, and XIII, which may be due to the loss of these proteins during the evolution of peach. This finding indicates that some non-conserved bHLH subfamilies among certain plant species may have specifically evolved to meet the developmental needs of the plant or for stress resistance. The number of peach bHLH proteins within each subfamily varied from 1 to 12. Most importantly, we found that some adjacent genes on the same chromosome were clustered together in the phylogenetic tree. For example, Prupe.5G130300.1, Prupe.5G130400.1, Prupe.5G130500.1, Prupe.5G130600.1, and Prupe.5G130700.1 were clustered into the branch of subfamily III d+e, and Prupe.8G166900.1 and Prupe.8G166800.1 were clustered together in subfamily III a. These results could be explained by the observation made in other plant species that members within the same clade might have recent common evolutionary origins and conserved molecular functions. The genes of a given bHLH subfamily of a plant are probably involved in the same pathway or biological process. Frequently, these proteins have overlapping functions, making them partially or totally redundant [6].

Some good examples of functional conservation of members within the same subfamily among different plant species have been provided. For example, *AtSPCH* (*AT5G53210*), *AtMUTE* (*AT3G06120*), and *AtFMA* (*AT3G24140*) and corresponding rice orthologs (*Os053SPC1*, *Os055MUTE*, and *Os051FMA*), which are members of subfamily I a, have been reported to function in controlling stomatal development [30,31]. In this study, *AT5G53210*, *AT3G06120*, and *AT3G24140* and the corresponding peach orthologs (*Prupe*.*2G307300*.*1*, *Prupe*.*1G151800*.*1*, *Prupe*.*1G006600*.*1*) were clustered within one clade of subfamily I a (Fig 1). Based on the functional conservation of orthologs within a given subfamily, we deduced that *Prupe*.*2G307300*.*1*, *Prupe*.*1G151800*.*1*, and *Prupe*.*1G006600*.*1* may also be associated with stomatal development. Three peach *bHLH* orthologs (*Prupe*.*7G132300*.*1*, *Prupe*.*1G434000*.*1*, and *Prupe*.*6G159200*.*1*) were found to cluster into the same clade of subfamily VIII b with four corresponding members from *Arabidopsis*, *At037HEC2* (*AT3G50330*), *At088HEC1* (*AT5G67060*), *At040IND* (*AT4G00120*), and *At043HEC3* (*AT5G09750*), which have been reported to play important roles in flower and fruit development [12]. Based on this observation, we deduced that *Prupe*.*7G132300*.*1*, *Prupe*.*1G434000*.*1*, and *Prupe*.*6G159200*.*1* likely coordinately regulate the development of peach flowers and fruits. In tomato, five genes (*SlbHLH073*, *SlbHLH078*, *SlbHLH008*, *SlbHLH127*, and *SlbHLH069*) belonging to subfamily IX have been inferred to be involved in fruit development and fruit ripening based on the expression profile [9]. Furthermore, *SlbHLH09* has been previously demonstrated to play a role in fruit ripening [32]. Thus, peach *bHLHs* in subfamily IX might also be associated with fruit ripening. Additionally, the *AtbHLHs* of subfamily VIII a have been found to be functionally replaceable by the *bHLHs* of subfamily VIII a of *Marchantia polymorpha*, which have been demonstrated to function as core regulators of reproductive development [33]. Therefore, peach *bHLHs* in subfamily VIII a may also play key roles in the reproductive process.

In subfamily I b, we found that six peach *bHLHs* (*Prupe*.*7G040300*.*1*, *Prupe*.*7G040500*.*1*, *Prupe*.*7G040100*.*1*, *Prupe*.*7G039900*.*1*, *Prupe*.*7G039800*.*1*, *Prupe*.*1G213700*.*1*) were clustered together with four *AtbHLHs* (*AtbHLH38/AT3G56970*, *AtbHLH39/AT3G56980*, A*tbHLH100/AT2G41240*, *AtbHLH101/AT5G04150*), which are involved in iron deficiency responses and homeostasis [34]. Tomato also exhibits four *SlbHLHs* (*SlbHLH025*, *SlbHLH066*, *SlbHLH067*, *SlbHLH068*) that cluster correspondingly with five *AtbHLHs* (*AtbHLH47*, *AtbHLH38*, *AtbHLH39*, *AtbHLH100*, *AtbHLH101*) and are predicted to show a similar function to the five *AtbHLHs* in relation to iron deficiency and uptake based on their expression in roots [9]. These results imply that the six peach *bHLHs* (*Prupe*.*7G040300*.*1*, *Prupe*.*7G040500*.*1*, *Prupe*.*7G040100*.*1*, *Prupe*.*7G039900*.*1*, *Prupe*.*7G039800*.*1*, and *Prupe*.*1G213700*.*1*) may also function in the response to iron deficiency and uptake.

*CmbHLH2*, which clusters into subfamily III f, has been demonstrated to be positively correlated with the anthocyanin content of cultivars with red, pink and yellow flowers [19]. In this study on peach, *Prupe*.*8G242100*.*1*, the best ortholog of *CmbHLH2*, was also found to be located within the subfamily III f (Fig 1). Similarly, *AmDEL*, one of *bHLH* superfamily genes of *Antirrhinum majus*, has been reported to induce anthocyanin biosynthesis in the hairy roots of transformed tobacco and *Ipomea tricolor* [35]. A Blast search against the GDR database using the CDS sequence of *AmDEL* revealed that the best ortholog of *AmDEL* in peach was *Prupe*.*5G100700*.*1*, which is the other member of the two members of subfamily III f (Fig 1). These findings suggested that *Prupe*.*8G242100*.*1* and *Prupe*.*5G100700*.*1* of subfamily III f may be involved in the regulation of anthocyanin biosynthesis. Therefore, additional experiments are needed to explore and verify their functions in peach.

**Fig 2:**
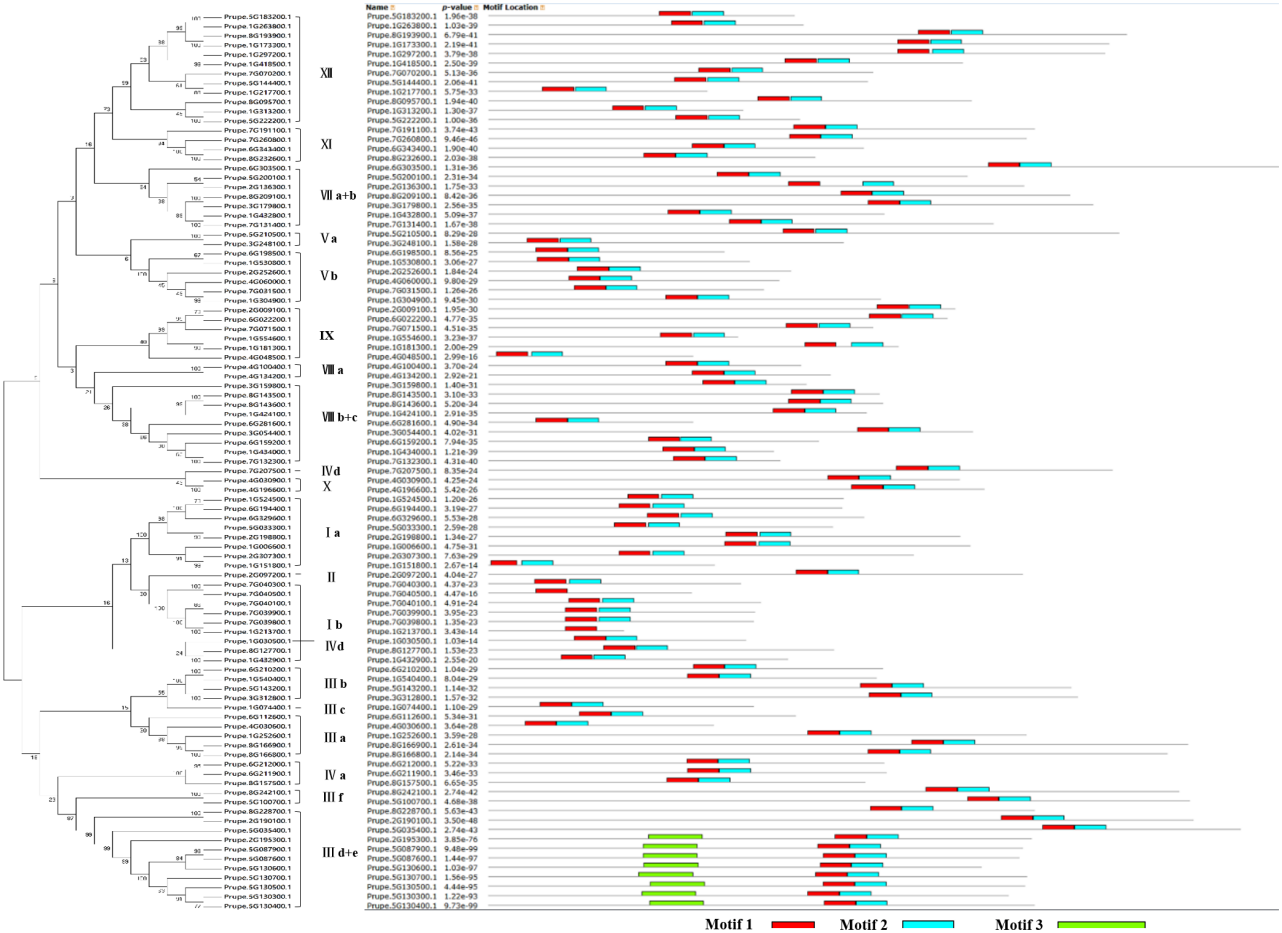
Motif distribution of peach bHLH superfamily proteins. The motifs of the bHLH superfamily proteins were analyzed using the MEME web server. The red, blue, and green blocks represent motifs 1, 2 and 3, respectively. The length of the gray line indicates the length of a sequence relative to all the other sequences. The position of each block indicates the location of a motif with a matching sequence. The phylogenetic tree on the right was constructed using only the sequences of bHLH proteins from peach.

**Fig 3:**
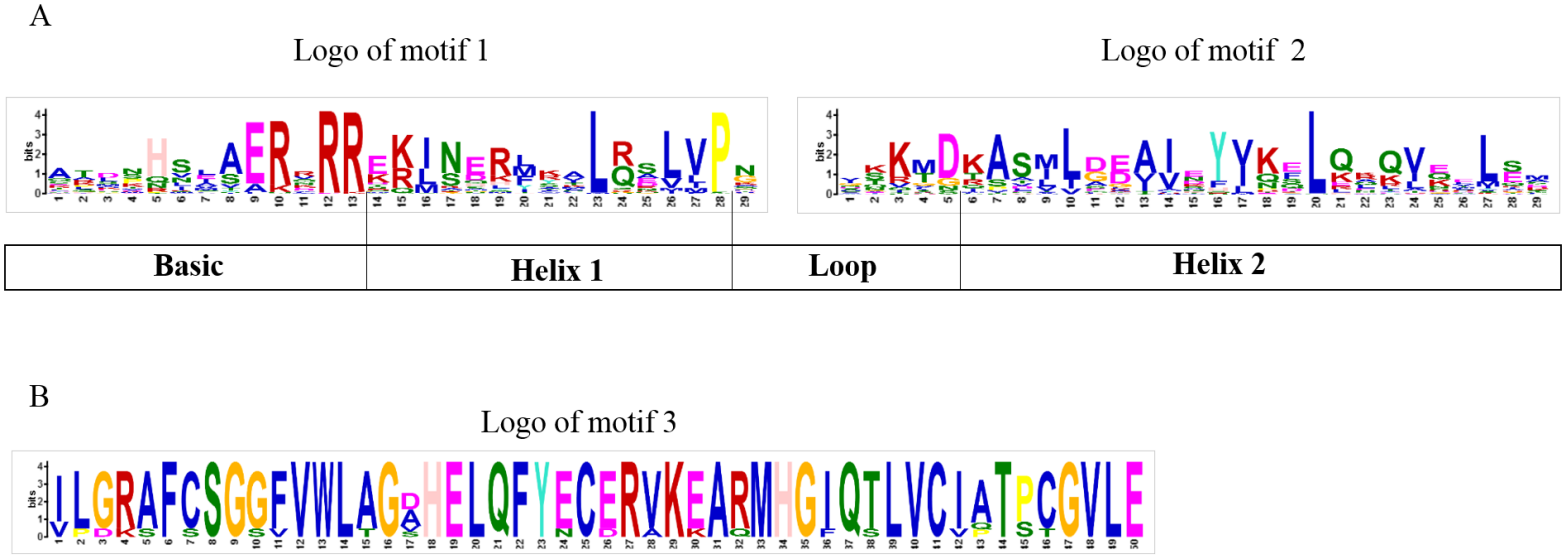
Motif composition and logos of peach bHLH proteins. A. The logos of motifs 1 and 2, which together constitute the bHLH domain in peach. The overall height of each stack represents the conservation of the sequence at that position. The capital letters indicate over 50% conservation of amino acids among the bHLH domains. The Arabic numerals under the colored capital letters indicate the width of the motif. Each color of the English letters represents a type of amino acid residue. B. The logo of motif 3, which constitutes another conserved motif, is present only in eight members of subfamily III e.

Additionally, to combine the phylogenetic tree and the features of the subfamily members, a phylogenetic tree was constructed using only the 95 sequences of the peach bHLH proteins (Fig 2). Although the ordering of the subfamilies in the phylogenetic tree is slightly different from that in the phylogenetic tree circled above (Fig 1), the member composition of each subfamily is the same as in the phylogenetic tree of the peach bHLH proteins circled above. The online MEME tool (version 4.8.1) was used to search for conserved motifs shared by bHLH proteins by uploading the 95 amino acid sequences of the peach bHLH superfamily [36]. Almost all the sequences exhibited two highly conserved motifs with the same width (29 amino acids), which are shown in the red and blue blocks, respectively (Fig 2 and S3 Table). Most of the motifs are located near the C-terminus of the peach bHLH proteins, such as the peach bHLH proteins in subfamilies III d+e, III a, III f, and IV a. Some motifs are located near the N-terminus, such as in the peach bHLH protein subfamily V a. The two motifs are very close to each other in 63 of the peach bHLH proteins but are slightly farther apart in a few of the peach proteins. Among the 95 peach bHLH proteins, the distance between the two motifs is widest in Prupe.2G236300.1. Based on a review of the literature regarding the bHLH domains of wheat [27] and tomato [9], in the present study, motif 1 (red block) was found to be composed of basic residues and helix 1, and motif 2 (blue block) was found to be composed of a loop and helix 2. In some bHLH proteins, the space between motifs 1 and 2 consists of a loop of variable length. Motif 1 (in red) and motif 2 (in blue) are composed of logos 1 and 2, respectively (Figs 2 and 3, S3 Table). The backbones of motifs 1 and 2 are also conserved at the amino acid level in the majority of plant species reported to date [9,27,37]. It has been deduced that the highly conserved residues in bHLH domains are responsible for dimerization [37].

In addition to these two common conserved motifs shared by the 95 peach bHLH proteins, the eight peach bHLH proteins (from Prupe.5G130300.1 to Prupe.2G195300.1) of subfamily III e from the phylogenetic tree harbor another highly conserved motif with a length of 50 amino acids. This motif is indicated by the green block and is composed of logo 3 (Fig 3 and S3 Table). This result is consistent with previous reports that members of a given subfamily of the bHLH superfamily exhibit another conserved motif (conserved non-bHLH motif) in addition to the common bHLH motif of plants [6]. In contrast, the existence of a conserved non-bHLH motif in addition to the bHLH domain strongly illustrates the correction of the subfamily classification based on the phylogenetic tree generated in this study. The conservation of motif 3 during peach evolution suggests that this motif must be essential for the function of the eight bHLH proteins in this subfamily. In addition, among the 95 bHLH proteins, Prupe.7G040500.1 and Prupe.1G213700.1 exhibited incomplete bHLH domains, whereas the remaining 93 bHLH proteins all presented complete bHLH domains. Similar observations have been made in other plant species, such as blueberry [11].

**Fig 4:**
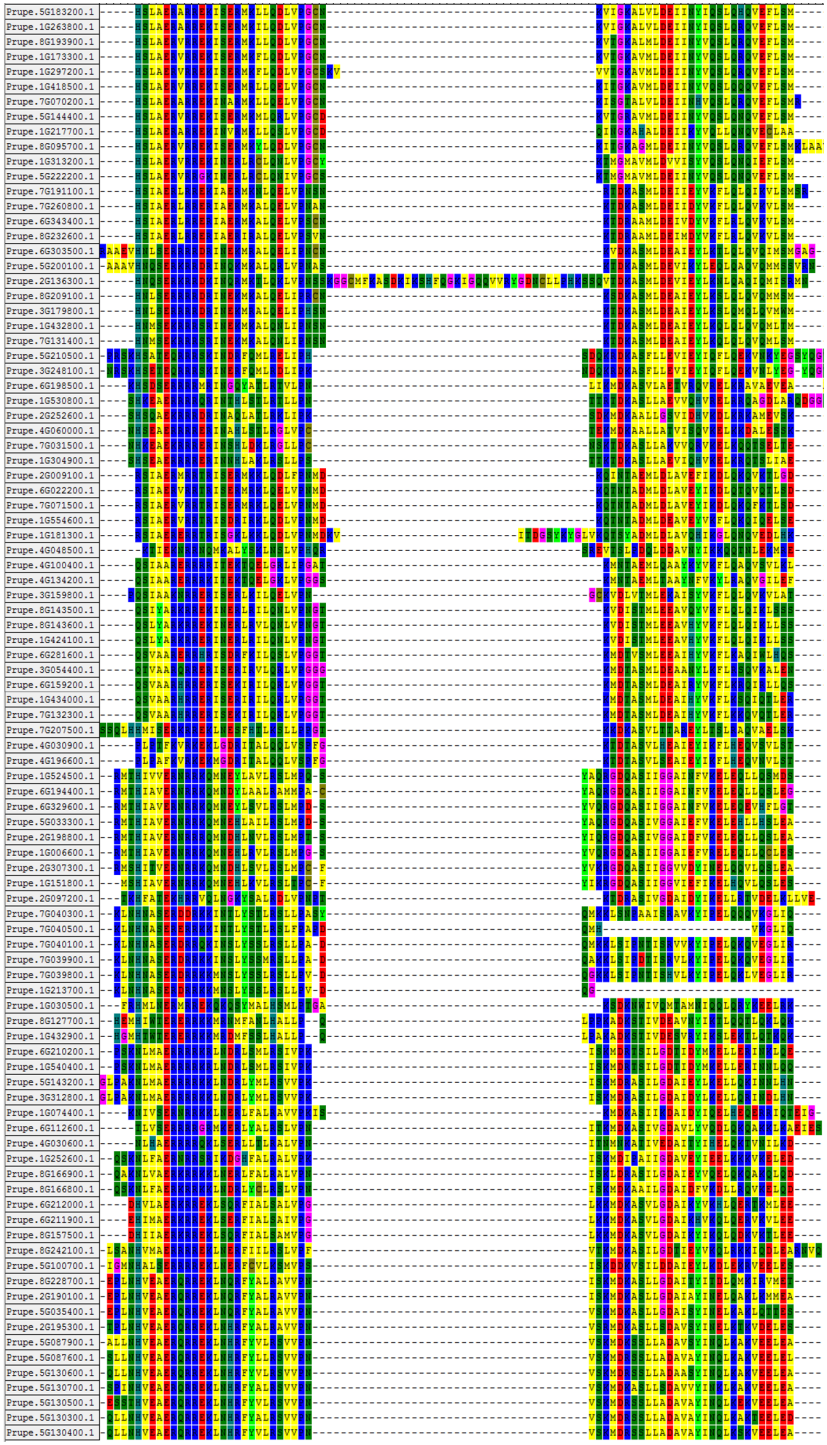
Multiple sequence alignment of the peach bHLH proteins. Amino acids with more than 50% identity are labeled with colored boxes. The ID order of 95 peach bHLH proteins is the same as in Fig 3.

Additionally, to analyze the sequence features of the bHLH domains at the amino acid level, we performed multiple sequence alignment using the sequences of the 95 bHLH proteins (Fig 4). There were two conserved regions in the sequences of the bHLH domain: the basic region plus helix 1 and the loop region plus helix 2. The results of multiple sequence alignment also confirmed the correction of the result obtained using the MEME tool described above. For example, a long amino acid sequence was found between the two highly conserved regions (Fig 4), which is consistent with the long distance between the two motifs in Prupe.2G136300.1, as shown in Fig 2. Additionally, the empty spaces in the second conserved region due to the absence of certain amino acids in the protein sequences of Prupe.7G040500.1 and Prupe.1G213700.1 (Fig 4) is consistent with the fact that Prupe.7G040500.1 and Prupe.1G213700.1 do not contain motif 2, as shown in Fig 2. Generally, the sequences of the basic region and the two helix regions were more conserved than the sequence of the loop region. These results obtained using MEME and sequence alignment tools further confirm that the results obtained from the HMM are highly reliable. These results also illustrate that the HMM is able to make highly accurate predictions for a given superfamily.

### Characteristics and structures of *bHLH* genes

**Fig 5:**
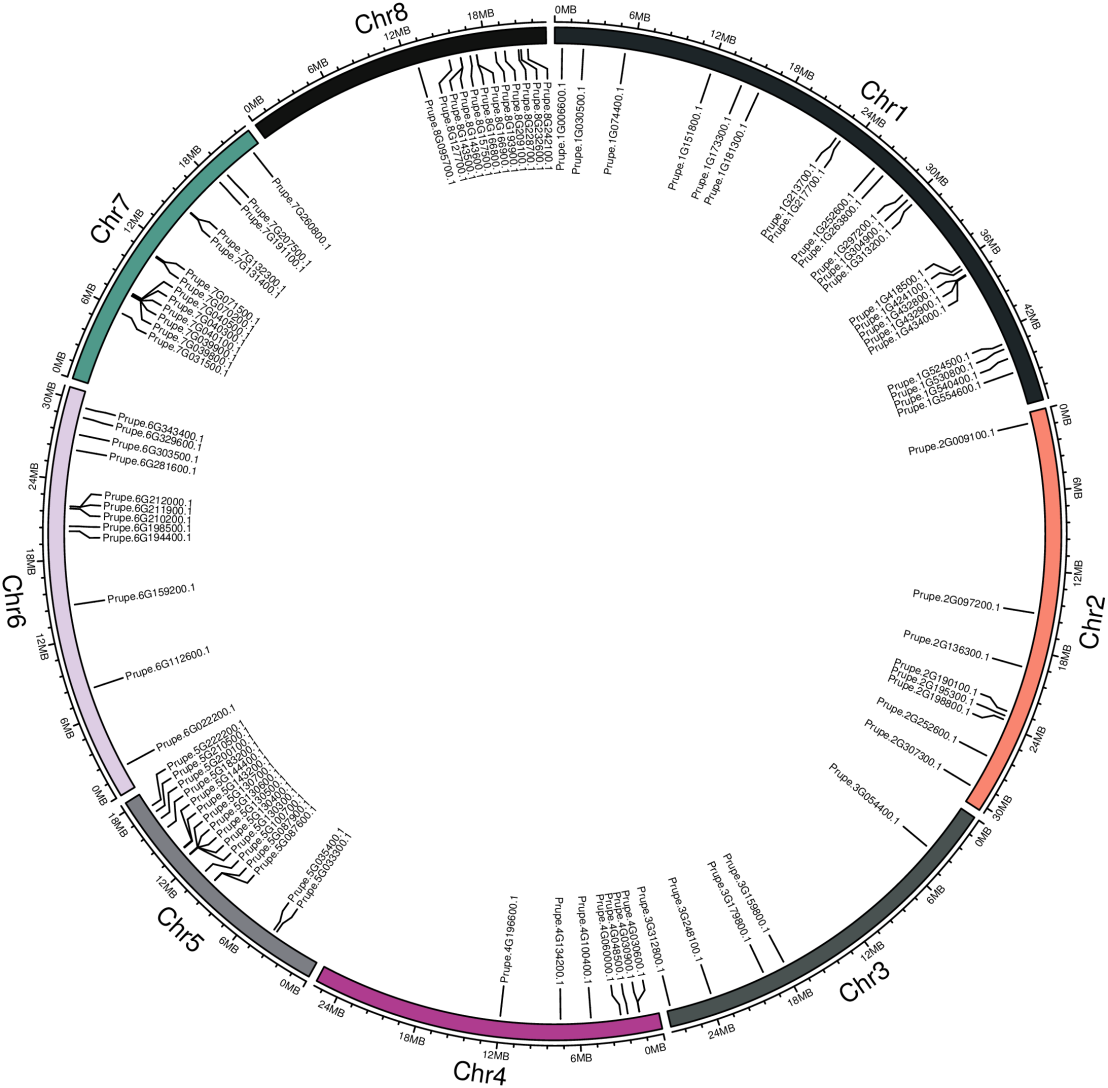
Distribution of *bHLH* superfamily genes on the eight chromosomes of peach.

Peach has eight chromosomes. Based on the data (S2 Table) regarding the distribution of peach *bHLH* genes on the chromosomes and the length of each chromosome obtained from the database, the chromosomal location of each *bHLH* gene was mapped to each chromosome using software to display the position intuitively. The 95 *bHLH* genes were found to be unevenly distributed on chromosomes 1 to 8 of the peach genome. The numbers of *bHLH* genes distributed on chromosomes 1 to 8 were 22,8,5,7,16,12,13, and 12, respectively (S2 Table and Fig 5). Chromosome 1 is the longest among the eight chromosomes in peach and exhibited the greatest number of *bHLH* genes. The 22 and 12 *bHLH* genes on chromosomes 1 and 6, respectively, were relatively well distributed. In contrast, the 12 and seven *bHLH* genes on chromosomes 8 and 4, respectively, were clustered near one of the two termini. Prupe.5G130300.1, Prupe.5G130400.1, Prupe.5G130500.1, Prupe.5G130600.1, and Prupe.5G130700.1 were not only clustered together, as shown in Fig 1, but were also located next to each other on chromosome 5 (Fig 5).

**Fig 6:**
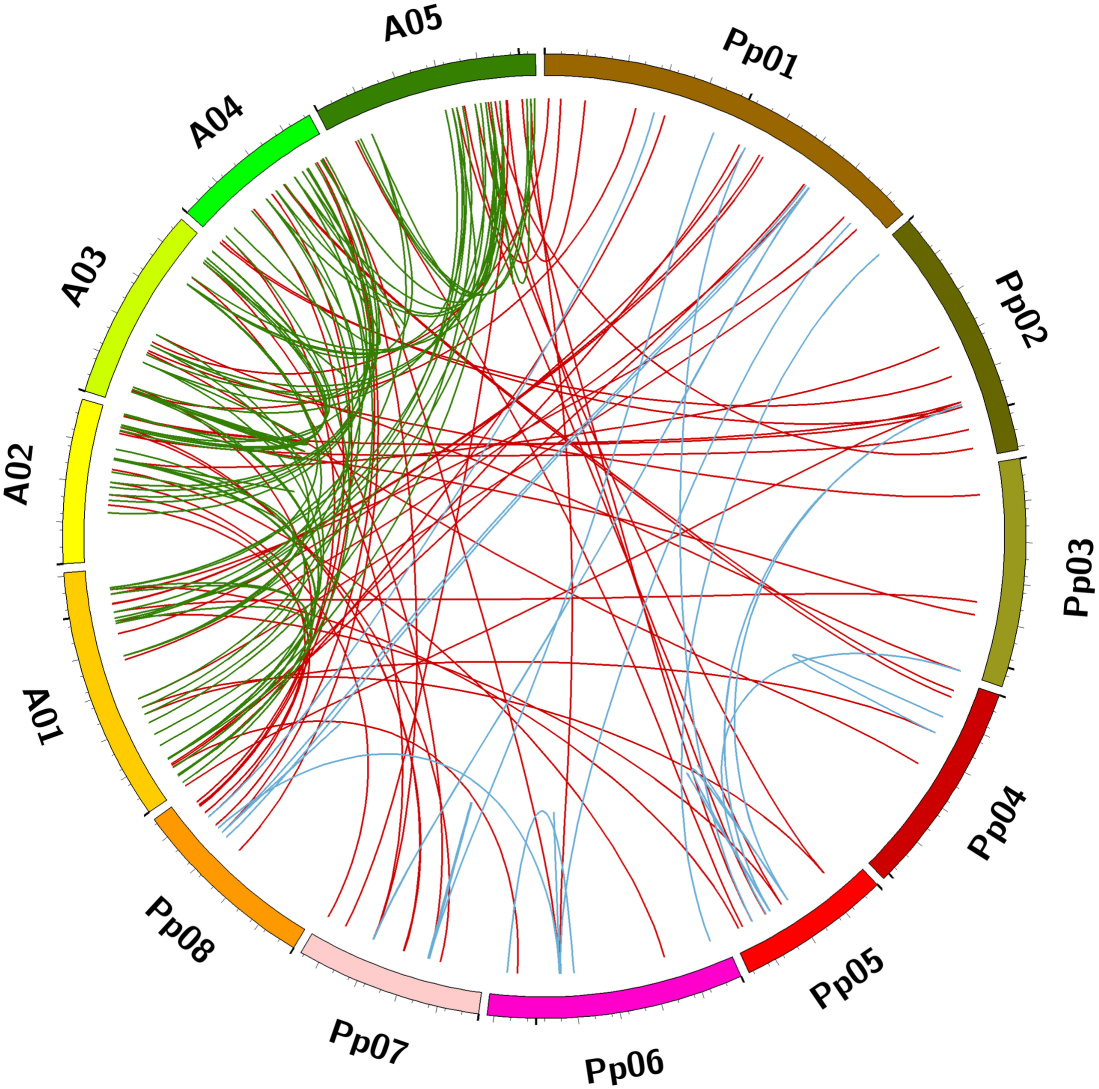
Syntenic relationships of *bHLH* superfamily genes in peach and *Arabidopsis*. Eight chromosomes of peach (Pp01–Pp08) and five chromosomes of *Arabidopsis* (A01–A05) are mapped in different colors. The red lines connect the orthologous *bHLH* genes between peach and *Arabidopsis*. The blue and green lines connect the paralogous *bHLH* genes in peach and *Arabidopsis*, respectively.

To further intuitively display and illustrate the genetic divergence and gene duplication within the bHLH superfamily, homology comparisons and syntenic relationships were analyzed to identify orthologous *bHLH* genes between peach and *Arabidopsis* as well as paralogous *bHLH* genes in peach or *Arabidopsis* using the OrthoMcl and Circos tools. As a result, 57 pairs of orthologous *bHLH* genes between peach and *Arabidopsis* were identified among the 95 *bHLH* genes of peach and 161 *bHLH* genes of *Arabidopsis* (Fig 6 (red line) and S4 Table). Among the orthologous gene pairs between peach and *Arabidopsis*, we found that each *Arabidopsis bHLH* gene shared only one orthologous gene with peach. This observation is not consistent with the findings reported in cabbage, where each *Arabidopsis bHLH* gene presents one to three orthologous genes in Chinese cabbage [13]. Nine of the 13 *bHLH* genes on chromosome 1 of peach were orthologous to nine *bHLH* genes on chromosome 1 of *Arabidopsis*, and the remaining four peach *bHLH* genes were orthologous to two *bHLH* genes on each of chromosomes 3 and 5 of *Arabidopsis* (A03 and A05). Four of the eight *bHLH* genes on chromosome 2 of peach were orthologous to four *bHLH* genes on chromosome 2 of *Arabidopsis*, and the remaining four peach *bHLH* genes were orthologous to four *bHLH* genes on chromosomes 1, 4, and 5 of *Arabidopsis*. Among the *bHLH* orthologous genes on chromosome 3 of peach, 50% exhibited orthologs on chromosome 3 of *Arabidopsis*. The same situation was observed for peach chromosome 4. Five of the eight *bHLH* genes on chromosome 5 of peach were orthologous to *bHLH* genes on chromosome 5 of *Arabidopsis*, and the remaining three were orthologous to *bHLH* genes on chromosome 1 of *Arabidopsis* (Fig 6 (red line) and S4 Table). Four orthologous *bHLH* genes on chromosome 6 of peach were orthologous to corresponding *bHLH* genes on chromosome 1, 2, 4, and 5 of *Arabidopsis*. Half of the orthologous *bHLH* genes on chromosome 7 of peach were orthologous to *bHLH* genes on chromosome 2 of *Arabidopsis*. Five of the eight *bHLH* genes on chromosome 8 of peach were orthologous to five *bHLH* genes on chromosome 4 of *Arabidopsis*. These results indicated that the *bHLH* genes on chromosome 1 of peach are more homologous to *bHLH* genes on the corresponding chromosome 1 of *Arabidopsis* than genes on the other *Arabidopsis* chromosomes. The same tendency was observed for the *bHLH* genes on chromosomes 2 to 5 of peach. These findings indicated that orthologous genes are evolutionarily conserved in land plants. Most orthologous genes of one plant species might evolve and originate from the corresponding chromosome of the ancestor plant. Additionally, *bHLH* genes on chromosomes 7 and 8 of peach shared higher homology with *bHLH* genes on chromosomes 2 and 4 of *Arabidopsis*, respectively, than genes on other *Arabidopsis* chromosomes. However, the orthologous *bHLH* genes on chromosome 6 of peach did not exhibit this characteristic but rather presented orthologs distributed on four of the five chromosomes of *Arabidopsis*. Hence, the mechanism underlying the evolution of these syntenic pairs on different or corresponding chromosomes between *Arabidopsis* and peach remains unclear. These genes may be subjected to several types of evolutionary selection. Because integration of the multiple aspects of gene evolution is very complex, it is impossible to summarize based on a single factor.

In addition, 48 pairs of paralogous *bHLH* genes were identified on eight chromosomes of peach (linked by blue lines in Fig 6; S5 Table). Each gene exhibited one to seven paralogous *bHLH* genes in peach; for example, *Prupe*.*5G087600*.*1* and *Prupe*.*2G195300*.*1* presented six and seven paralogous *bHLH* genes, respectively. In fact, the 48 pairs of paralogous *bHLH* genes in peach were found to be composed of 40 peach *bHLH* genes. Regarding paralogous *bHLH* genes in peach (blue line in Fig 6), no paralogous *bHLH* genes were found to exist on the two adjacent peach chromosomes. In contrast, we observed that among the 161 *bHLH* genes of *Arabidopsis*, 81 pairs of paralogous *bHLH* genes were present on five chromosomes (shown with green lines in Fig 6; S5 Table). Similar to peach, each gene exhibited one to six paralogous *bHLH* genes in *Arabidopsis*; for example, *AT5G54680* and *AT4G25410* presented six and five paralogous *bHLH* genes, respectively. This feature has also been observed in other studies [13,27]. Similar to peach, the 81 pairs of paralogous *bHLH* genes in *Arabidopsis* are composed of 88 *Arabidopsis bHLH* genes. Consistent with a previous study in *Brachypodium distachyon* [2], most of the paralogous *bHLH* genes in peach were grouped within the same subfamily as the original genes. For example, the seven *bHLH* genes paralogous to *Prupe*.*2G195300*.*1* were clustered in subfamily III d+e (Fig 1 and S2 Table). Based on these results, we deduced that the genes of the bHLH superfamily likely shared a common origin and gradually expanded under intense selective pressure approximately 4–14 Mya, leading to the functional conservation and divergence of the bHLH superfamily observed in peach.

**Fig 7:**
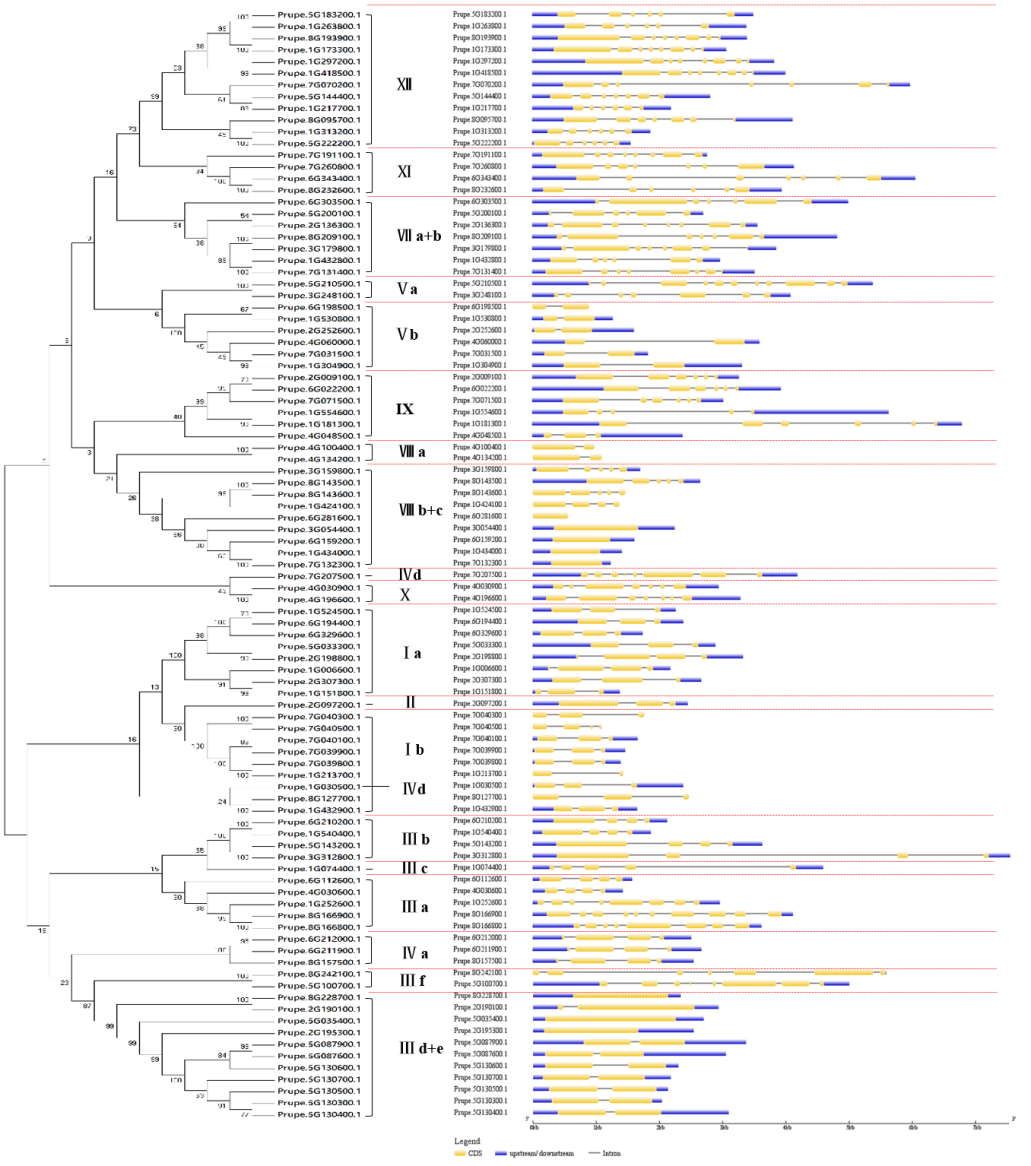
Locations and lengths of the exons and introns of peach *bHLH* superfamily genes. Exons and introns are presented as filled yellow sticks and thin gray single lines, respectively. UTRs are represented by dark blue bars at the ends. The red dotted line separates each subfamily and clearly presents the member conservation of each subfamily. The phylogenetic tree on the right was constructed using only the sequences of bHLH proteins in peach.

The schematic structure of peach *bHLH* genes was analyzed using the GSDS tool (Fig 7). *Prupe*.*5G130400*.*1*, *Prupe*.*5G130300*.*1*, P*rupe*.*5G130500*.*1*, *Prupe*.*5G130700*.*1*, *Prupe*.*5G130600*.*1*, *Prupe*.*5G087600*.*1*, and *Prupe*.*5G087900*.*1* of subfamily III d+e were observed to exhibit two exons and one intron and to be clustered within a single branch of the phylogenetic tree (Fig 1). *Prupe*.*6G212000*.*1*, *Prupe*.*6G211900*.*1*, and *Prupe*.*8G157500*.*1* of subfamily IV exhibited four exons and three introns and were also clustered within a single branch of the phylogenetic tree (Fig 1). *Prupe*.*6G210200*.*1*, *Prupe*.*1G540400*.*1*, *Prupe*.*5G143200*.*1*, and *Prupe*.*3G312800*.*1* of subfamily III b presented four exons and three introns and were clustered within a single clade in the phylogenetic tree (Fig 1). Members of subfamily I also exhibited three exons and two introns. The length and location of each exon in the genome sequence of these four genes were also highly conserved among the four genes (Fig 7). The members of subfamilies III a, V a, VII a+b, IV d, IX, X, XI, and XII presented relatively greater numbers of exons than the other subfamilies in this study. Overall, the numbers, lengths, and positions of the exons were relatively well conserved in each subfamily of peach *bHLH* genes. This finding is consistent with previous reports about both *bHLH* genes in other plant species [2] and other TF families [38,39]. Exon/intron organization is considered to play an important role in the evolution of the genes of a given family [40].

### Expression analysis of peach *bHLH* superfamily genes

**Fig 8:**
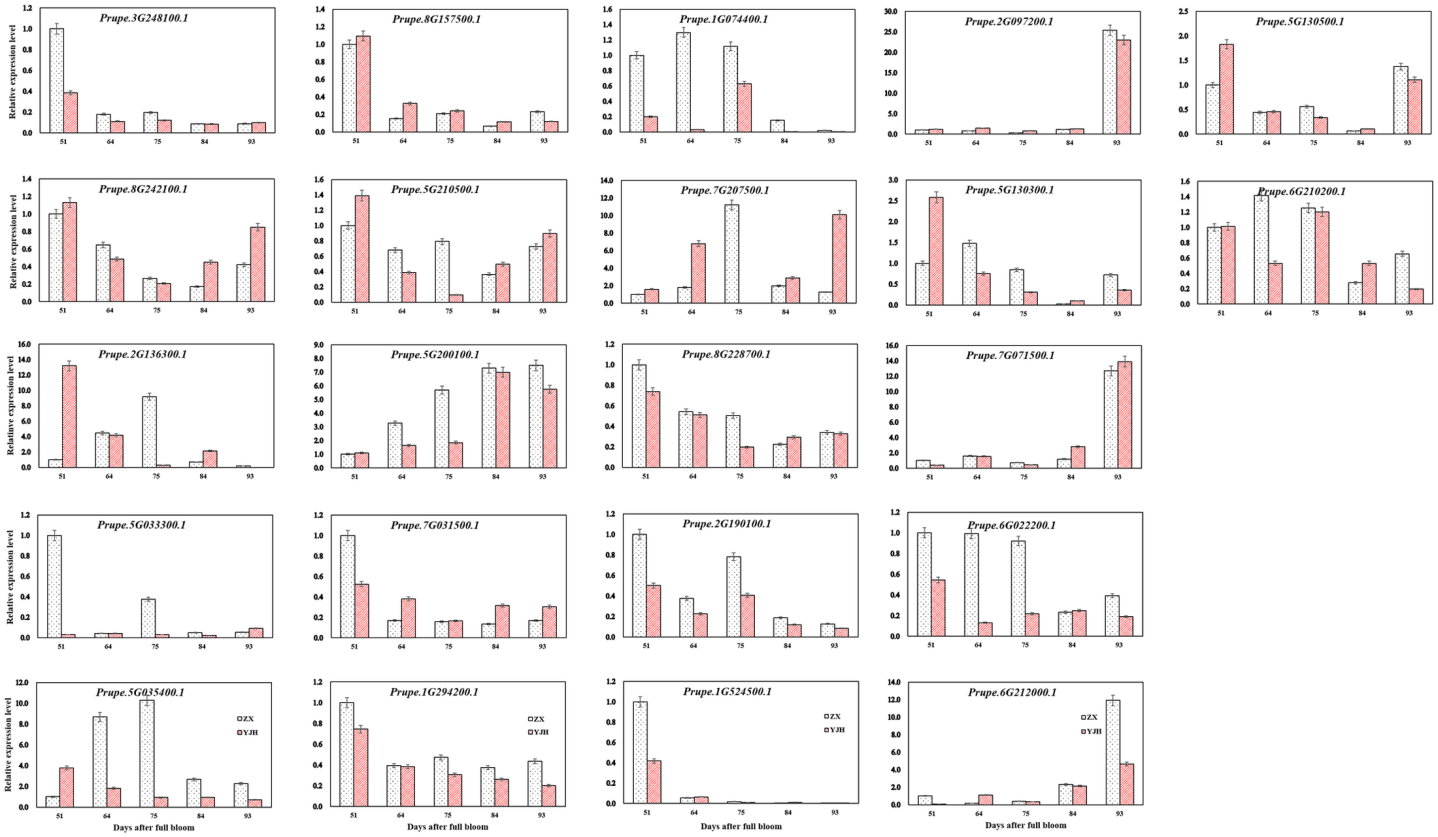
Relative expression levels of selected *bHLH* genes in fruits of the two peach cultivars. Error bars represent standard errors from three independent replicates.

To assess the potential regulatory role of *bHLH* superfamily genes in peach fruits, the expression levels of 22 *bHLH* superfamily genes in the white-fleshed fruit of ZX and red-fleshed fruit of YJH sampled 51, 64, 75, 84, and 93 DAFB were analyzed in this study (Fig 8). The expression of *Prupe*.*1G524500*.*1* in the fruits of ZX and YJH decreased significantly, by approximately 1- and 0.4-fold compared with the initial level, and was almost undetectable at 93 DAFB in the fruits of both cultivars. At 51 DAFB, the expression level of *Prupe*.*1G524500*.*1* in the fruit of ZX was lower than that in the fruit of the YJH cultivar. The expression of *Prupe*.*1G294200*.*1* in the fruit of YJH decreased gradually with development from young to harvested fruit. In contrast, in the ZX cultivar, no significant difference in the expression level of *Prupe*.*1G294200*.*1* was observed from 64 to 93 DAFB. However, from 51 to 64 DAFB, there was a sharp decrease in the expression level of *Prupe*.*1G294200*.*1*. A similar expression pattern to that found for *Prupe*.*1G294200*.*1* was observed for *Prupe*.*8G228700*.*1* in both cultivars. The expression of *Prupe*.*5G035400*.*1* in the fruit of YJH decreased gradually as the fruit matured, whereas the expression level of *Prupe*.*5G035400*.*1* in the fruit of ZX increased from 51 to 75 DAFB and then decreased until harvest. A similar expression pattern to that of *Prupe*.*5G035400*.*1* was observed for *Prupe*.*2G136300*.*1* in both cultivars. A sharp decrease in the expression of *Prupe*.*7G031500*.*1* from 51 to 64 DAFB followed by relatively stable expression from 64 to 93 DAFB was observed in the ZX cultivar. The expression level of *Prupe*.*3G248100*.*1* decreased gradually from 51 to 93 DAFB in both cultivars. The expression level of *Prupe*.*3G248100*.*1* in ZX was relatively higher than that in YJH from 51 to 75 DAFB. The expression level of *Prupe*.*3G248100*.*1* was almost equal in ZX and YJH from 84 to 93 DAFB. A trend consisting of a gradual decrease in expression followed by a gradual increase was observed for *Prupe*.*8G242100*.*1* in both cultivars. *Prupe*.*5G210500*.*1* exhibited a similar expression pattern to *Prupe*.*5G033300*.*1*, except at 75 DAFB in ZX. *Prupe*.*5G033300*.*1* presented very low and relatively stable expression throughout the development of YJH, whereas in the young fruit of ZX, *Prupe*.*5G033300*.*1* exhibited relatively high levels of expression. The expression of *Prupe*.*5G200100*.*1* increased gradually with fruit development from 51 to 93 DAFB in both cultivars, except at 93 DAFB in YJH. Overall, the expression level of *Prupe*.*5G200100*.*1* in ZX was higher than that in YJH, and the expression level of *Prupe*.*5G200100*.*1* remained stable in ZX from 84 to 93 DAFB. Surprisingly, the variable expression trend of *Prupe*.*2G190100*.*1* was the same in both cultivars, and the expression level of *Prupe*.*2G190100*.*1* in ZX was always higher than that in YJH. *Prupe*.*5G033300*.*1* and *Prupe*.*1G524500*.*1*, both of which belong to subfamily I a, exhibited relatively similar expression patterns in this study. This finding illustrates that members of the same bHLH subfamily might have redundant functions, although this hypothesis requires further verification. These proteins probably participate in the same network to regulate certain biological or biochemical processes simultaneously. *Prupe*.*7G071500*.*1*, *Prupe*.*2G097200*.*1*, and *Prupe*.*6G212000*.*1* presented relatively low expression levels from 51 to 84 DAFB but displayed high expression levels in the harvested mature fruits of both cultivars.

The different expression patterns involving gradual increases and decreases observed in this study illustrate that some *bHLH* superfamily genes may be associated with fruit development. This finding was consistent with previous findings that bHLH proteins play a regulatory role in fruit ripening in peach and other fruit species [41]. Additionally, the findings of this study are in agreement with transcriptomic results [42], which revealed that the transition from developmental stage 3 to stage 4 is accompanied by up-regulated expression of some *bHLH* genes in peach [36]. Similarly, it has been previously reported that *bHLH3* together with *MYB* and *WD40* can form a regulatory complex to regulate flavonoid biosynthesis by activating the transcription of downstream genes in nectarine [43]. Similar results have been observed for anthocyanin accumulation in apple [44] and peach [45] fruits, where MYB TFs use bHLH proteins as a partner to activate the transcription of anthocyanin pathway genes. Montefiori et al. [46] reported that bHLH TFs are involved in the regulation of the flavonoid pathway in a variety of species. Liu et al. showed that significantly increased anthocyanin accumulation in peels is accompanied by enhanced expression of *bHLH3* [47]. In rice, it has been reported that bHLH proteins are involved in controlling grain length and weight [48]. The results of the present study indicated that *Prupe*.*8G242100*.*1*, *Prupe*.*7G031500*.*1*, and *Prupe*.*7G207500*.*1* may be associated with anthocyanin biosynthesis, which needs to be further confirmed experimentally. Gene expression is one of the vital regulatory mechanisms utilized by plant cells to carry out their functions. During fruit development and ripening, fleshy fruits undergo a series of complex biochemical reactions, in addition to physiological and dynamic changes. These internal and environmental factors collectively affect the phenotypes of fruits, including their acidity or sweetness, coloration, hardness or softness, flavor, and aroma, increasing the nutritional value and attractiveness of the fruits [36].

## Conclusions

This study constitutes the first comprehensive and systematic analysis of *bHLH* superfamily genes in peach based on the whole-genome sequence. As a result, a total of 95 *bHLH* superfamily genes were identified from the peach genome and were clustered into 19 subfamilies. The characterization of the genes and proteins, including their motif compositions and exon/intron organizations, indicated that the members within each subfamily are highly conserved. Additionally, some common bHLH subfamilies that exist in other plant species were also identified in peach, indicating that the highly conserved bHLH subfamilies among plant species may play fundamental roles in the growth and development of these plant species. The 95 *bHLH* genes of peach were found to be unevenly distributed on chromosomes 1 to 8 of the peach genome. Among these genes, there are 48 pairs of paralogous *bHLH* genes on the eight chromosomes of the peach genome. Additionally, 57 pairs of bHLH members were found to be orthologous between peach and *Arabidopsis*. The identification of peach *bHLH* genes homologous to *Arabidopsis* genes enriches the genome annotation and facilitates the prediction of the functions of *bHLH* superfamily genes in peach. The expression patterns observed in both white-fleshed and red-fleshed fruits in this study provide preliminary data for further analysis of *bHLH* genes associated with fruit development. This study lays the foundation for further functional verification of *bHLH* superfamily genes in peach and enriches the knowledge of *bHLH* superfamily genes in plant species.

## Supporting information

S1 Table Sequences of primers designed for qRT-PCR analysis of 22 selected *bHLH* superfamily genes. (XLSX)

S2 Table Summary of *bHLH* superfamily genes in peach. (XLSX)

S3 Table Sequences of conserved motifs shared by bHLH superfamily proteins in peach. (XLSX)

S4 Table Summary of orthologous *bHLH* genes between peach and *Arabidopsis*. Each pair of orthologous *bHLH* genes between peach and *Arabidopsis* is listed on the same line.
(XLSX)

S5 Table Summary of paralogous *bHLH* genes in *Arabidopsis* (green) or peach (blue). Each pair of paralogous *bHLH* genes in peach or *Arabidopsis* is listed on the same line. The order number is used for displaying repeated gene IDs.
(XLSX)

## Abbreviations

bHLH: basic helix-loop-helix
CDSs: coding sequences
DAFB: days after full bloom
GDR: Genome Database for Rosaceae
GSDS: Gene Structure Display Server
HMM: hidden markov model
MEME: Multiple EM for Motif Elicitation
ORF: the open reading frame
qRT-PCR: quantitative real-time polymerase chain reaction
RP II: RNA polymerase II
TAIR: The Arabidopsis Information Resource
TEF2: translation elongation factor 2
TFs: transcription factors
YJH: Yejihong
ZX: Zhaoxia
